# Machine‐learning analysis identifies “elite” viral controllers with increased survival and homeostatic responses in critical COVID‐19

**DOI:** 10.1002/ctm2.70241

**Published:** 2025-04-25

**Authors:** Nadia García‐Mateo, Alejandro Álvaro‐Meca, Tamara Postigo, Alicia Ortega, Amanda de la de la Fuente, Raquel Almansa, Noelia Jorge, Laura González‐González, Lara Sánchez Recio, Isidoro Martínez, María Martín‐Vicente, María José Muñoz‐Gómez, Vicente Más, Mónica Vázquez, Olga Cano, Daniel Vélez‐Serrano, Luis Tamayo, José Ángel Berezo, Rubén Herrán‐Monge, Jesús Blanco, Pedro Enríquez, Pablo Ryan‐Murua, Amalia de la Martínez de la Gándara, Covadonga Rodríguez, Gloria Andrade, Elena Bustamante‐Munguira, Gloria Renedo Sánchez‐Girón, Ramón Cicuendez Ávila, Juan Bustamante‐Munguira, Wysali Trapiello, Elena Gallego Curto, Alejandro Úbeda‐Iglesias, María Salgado‐Villén, Enrique Berruguilla‐Pérez, María del Carmen del de la Torre, Estel Güell, Fernando Casadiego, Ángel Estella, María Recuerda Núñez, Juan Manuel Sánchez Calvo, Sandra Campos‐Fernández, Yhivian Peñasco‐Martín, María Teresa García Unzueta, Ignacio Martínez Varela, María Teresa Bouza Vieiro, Felipe Pérez‐García, Ana Moreno‐Romero, Lorenzo Socias, Juan López Messa, Leire Pérez Bastida, Pablo Vidal‐Cortés, Lorena del del Río‐Carbajo, Jorge del Nieto del Olmo, Estefanía Prol‐Silva, Víctor Sagredo Meneses, Noelia Albalá Martínez, Milagros González‐Rivera, José Manuel Gómez, Nieves Carbonell, María Luisa Blasco, David de de Gonzalo‐Calvo, Jessica González, Jesús Caballero, Carme Barberá, María Cruz Martín Delgado, Luis Jorge Valdivia, Caridad Martín‐López, María Teresa Nieto, Ruth Noemí Jorge García, Emilio Maseda, Ana Loza‐Vázquez, José María Eiros, Anna Motos, Laia Fernández‐Barat, Joan Casenco‐Ribas, Adrián Ceccato, Ferrán Barbé, David J. Kelvin, Jesús F. Bermejo‐Martin, Ana P. Tedim, Salvador Resino, Antoni Torres

**Affiliations:** ^1^ Group for Biomedical Research in Sepsis (BioSepsis) Instituto de Investigación Biomédica de Salamanca (IBSAL) Gerencia Regional de Salud de Castilla y León Salamanca Spain; ^2^ Centro de Investigación Biomédica en Red en Enfermedades Respiratorias (CIBERES) Instituto de Salud Carlos III Madrid Spain; ^3^ Department of Preventive Medicine and Public Health Faculty of Health Science Universidad Rey Juan Carlos Alcorcón Madrid Spain; ^4^ Centro de Investigación Biomédica en Red en Enfermedades Infecciosas (CIBERINFEC) Instituto de Salud Carlos III Madrid Spain; ^5^ Department of Cellular Biology Histology and Pharmacology University of Valladolid Valladolid Spain; ^6^ Viral Infection and Immunity Unit Centro Nacional de Microbiología Instituto de Salud Carlos III Majadahonda Spain; ^7^ Department of Statistics and Operations Research Universidad Complutense de Madrid Plaza de Ciencias Madrid Spain; ^8^ Critical Care Medicine Service Hospital Universitario Rio Hortega, Gerencia Regional de Salud de Castilla y León Valladolid Spain; ^9^ Internal Medicine Service Hospital Infanta Leonor Madrid Spain; ^10^ Critical Care Medicine Service, Hospital Infanta Leonor, Avenida de la Gran Vía del Este Madrid Spain; ^11^ Critical Care Medicine Service Hospital Clínico Universitario de Valladolid Gerencia Regional de Salud de Castilla y León Valladolid Spain; ^12^ Cardiovascular Surgery Service Hospital Clínico Universitario de Valladolid Gerencia Regional de Salud de Castilla y León Valladolid Spain; ^13^ Clinical Analysis Service Hospital Clínico Universitario de Valladolid Gerencia Regional de Salud de Castilla y León Valladolid Spain; ^14^ Critical Care Medicine Service Hospital San Pedro de Alcántara Cáceres Spain; ^15^ Critical Care Medicine Service Hospital Punta de Europa Algeciras Spain; ^16^ Unidad de Gestión Clínica de Análisis Clínicos Hospital Punta de Europa Algeciras Spain; ^17^ Department of Intensive Care Medicine Hospital de Mataró Mataró Barcelona Spain; ^18^ Intensive Care Unit Hospital Universitario de Jerez Departamento de Medicina Universidad de Cádiz INiBICA Jerez de la Frontera Spain; ^19^ Microbiology Department Hospital Universitario de Jerez Jerez de la Frontera Spain; ^20^ Critical Care Medicine Service Hospital Universitario Marqués de Valdecilla Santander Spain; ^21^ Servicio de Análisis Clínicos Hospital Universitario Marqués de Valdecilla Santander Spain; ^22^ Critical Care Department Hospital Universitario Lucus Augustí Lugo Spain; ^23^ Clinical Microbiology Service Hospital Universitario Príncipe de Asturias Madrid Spain; ^24^ Biomedicine and Biotechnology Department Faculty of Medicine Universidad de Alcalá Madrid Spain; ^25^ Clinical Analysis Service Hospital Universitario Príncipe de Asturias Madrid Spain; ^26^ Intensive Care Unit Hospital Universitario Son Llàtzer Palma Spain; ^27^ Critical Care Medicine Service Complejo Asistencial Universitario de Palencia Palencia Spain; ^28^ Intensive Care Unit Complejo Hospitalario Universitario de Ourense Ourense Spain; ^29^ Critical Care Medicine Service Complejo Universitario Asistencial de Salamanca Salamanca Spain; ^30^ Clinical Biochemistry Service Hospital General Universitario Gregorio Marañón Madrid Spain; ^31^ Critical Care Medicine Service Hospital General Universitario Gregorio Marañón Madrid Spain; ^32^ Intensive Care Unit, Hospital Clínico Universitario de Valencia Valencia Spain; ^33^ Translational Research in Respiratory Medicine University Hospital Arnau de Vilanova and Santa Maria Institut de Recerca Biomèdica de Lleida Lleida Spain; ^34^ Critical Care Medicine Service Hospital Universitari Arnau de Vilanova Lleida Spain; ^35^ Critical Care Medicine Service Hospital Universitari de Santa Maria Lleida Spain; ^36^ Intensive Care Unit Hospital Universitario de Torrejón Universidad Francisco de Vitoria Torrejón de Ardoz Madrid Spain; ^37^ Critical Care Medicine Service Hospital Universitario de León León Spain; ^38^ Critical Care Medicine Service Hospital General de Segovia Segovia Spain; ^39^ Intensive Care Department Hospital Nuestra Señora de Gracia Zaragoza Spain; ^40^ Anesthesiology and Reanimation Service Hospital Universitario de la Paz Madrid Spain; ^41^ Critical Care Medicine Service Hospital Universitario Nuestra Señora de Valme Sevilla Spain; ^42^ Microbiology Service Hospital Universitario Río Hortega Gerencia Regional de Salud de Castilla y León Valladolid Spain; ^43^ Department of Pulmonology Hospital Clinic de Barcelona Institut D Investigacions August Pi I Sunyer (IDIBAPS) Universidad de Barcelona Barcelona Spain; ^44^ Critical Care Center Institut d'Investigació i Innovació Parc Taulí (I3PT) Hospital Universitari Sagrat Cor Sabadell Spain; ^45^ Department of Microbiology and Immunology Faculty of Medicine Canadian Center for Vaccinology (CCfV) Dalhousie University Halifax NS Canada; ^46^ Laboratory of Immunity Shantou University Medical College Shantou Guangdong China; ^47^ Department of Medicine Faculty of Medicine Universidad de Salamanca Salamanca Spain

1

Dear Editor,

The outcome of COVID‐19 disease is strongly related to the interaction between the virus and the host immune response, which may become dysregulated in critically ill patients. This dysregulated response is characterized by elevated levels of inflammatory mediators, an overactivation of the innate immune system,^1^ lymphopenia,^2^ delayed antibody and interferon responses,^3^ and a massive dissemination of viral components into the blood,^4^ all of which contribute to severity and increased mortality.^5^, ^6^, ^7^ These immune and non‐immune parameters can be integrated into so‐called combitypes^8^ to identify subgroups of patients with different immune profiles and outcomes, helping to guide clinical strategies. In a previous study we used viral RNA levels in plasma to categorize a multicentre cohort of critically ill COVID‐19 patients into three subgroups with different mortality rate.^4^ In this study, we combined virological data (SARS‐CoV‐2 N1 RNA plasma load and N‐antigenemia) and 32 host response biomarkers to improve classification of critically ill COVID‐19 patients, with the objective to identify biological clues explaining survival.

We conducted a prospective cohort study in 785 critically ill COVID‐19 patients with a plasma EDTA sample collected at intensive care unit (ICU) admission. The detailed methods and the biological parameters measured are summarized in the Supporting Information. The biological characteristics of 90‐day survivors compared to non‐survivors (Table S1) indicated that non‐survivors were more likely to exhibit the presence of SARS‐CoV‐2 N antigen, along with higher viral RNA load in plasma, higher tissue damage (RNase P RNA), lower lymphocyte counts, and higher neutrophils levels. Additionally, non‐survivors exhibited increased concentrations of multiple biomarkers involved in endothelial dysfunction (angiopoietin 2, endothelin‐1, ICAM‐1 and VCAM‐1), inflammation (TNF‐α, IL‐15 and IL‐6), coagulation (D‐dimmer), chemotaxis (CXCL10, CCL2, and IL‐8), immunosuppression (IL‐10, PD‐L1, and IL1‐RA), T‐cell biology (CD27), apoptosis (Fas) and innate immune‐related proteins (EGF and SP‐D).

Based on these biological characteristics, XGBoost algorithm was employed to develop a model for predicting 90‐day mortality (AUROC of 0.80) (Supplementary Figure 1) and SHAP values were obtained to evaluate the influence of each biological feature on the outcome variable (Figure 1). Levels of SARS‐CoV‐2 N1 RNA was the parameter ranking the first to predict 90‐day mortality, following by endothelin‐1, IL‐15, IL‐8, neutrophils, IL‐6, TREM‐1, CCL2, CD27, SP‐D, myeloperoxidase, IL‐10, D‐dimer, PTX‐3, CXCL10, RNase P and VCAM‐1, suggesting that viral control, endothelial dysregulation, pro‐inflammatory mechanisms and chemotaxis are key biological functions in determining 90‐day mortality in critical COVID‐19 disease. On the contrary, high levels of the cytokine RANTES, anti‐SARS‐CoV‐2 S IgM and anti‐SARS‐CoV‐2 S IgG antibodies represented a protective factor against mortality.

**FIGURE 1:**
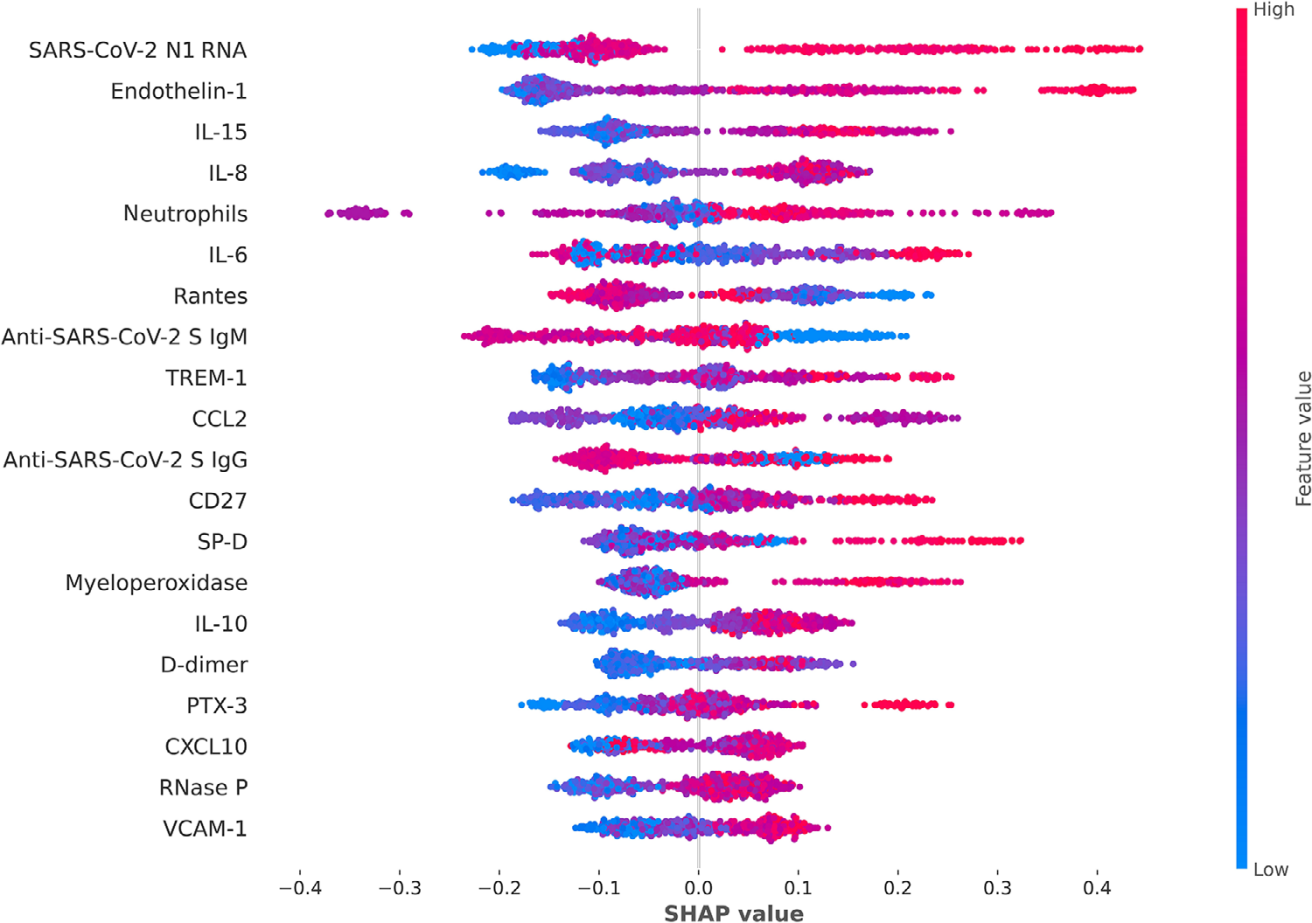
SHAP (SHapley Additive exPlanations) value distribution of the top 20 features obtained in the machine learning model for identifying the 90‐day mortality. Each point is a patient. The horizontal position of each point represents the SHAP value (importance higher or lower for prediction) and the direction of each feature for predicting a particular patient. Positive SHAP values predict true positives, and negative SHAP values predict true negatives. The red colour indicates high values, while the blue colour indicates low values of the features for a specific patient. Plasma biomarker values were ln‐transformed. SHAP values were calculated using the Python package XGBoost. Abbreviations: SARS‐CoV‐2, severe acute respiratory syndrome coronavirus 2; IL, interleukin; RANTES, regulated on activation, normal T cell expressed and secreted protein; IgG, immunoglobulin G; IgM, immunoglobulin M; TREM‐1, triggering receptor expressed on myeloid cells 1; CCL2, chemokine (C‐C motif) ligand 2; CD27, cluster differentiation 27 molecule; SP‐D, Surfactant protein D; PTX‐3, pentraxin 3; CXCL10, C‐X‐C motif chemokine ligand 10; VCAM‐1, vascular cell adhesion protein.

We further classified the patients into three groups or combitypes with different 90‐day mortality rate, using a partitional clustering method based on the biological characteristics (Figure 2A, B). The Combitype‐1 group was the most common (41.5%) and showed the lowest mortality rate at day 90 after ICU admission (7.7%), followed by the Combitype‐2 group (21.5%) with a 90‐day mortality rate of 25.4%. The 90‐day mortality dramatically increased to 65.9% in the Combitype‐3 group, who represented 36.9% of the cohort. Survival mean time in the first 90 days in each group was as follows [days (lower limit—upper limit)]: Combitype‐1 [84.7 (82.7–86.8)], Combitype‐2 [73.0 (68.5–77.6)] and Combitype‐3 [44.2 (40.2–48.2)] (Figure 2C).

**FIGURE 2:**
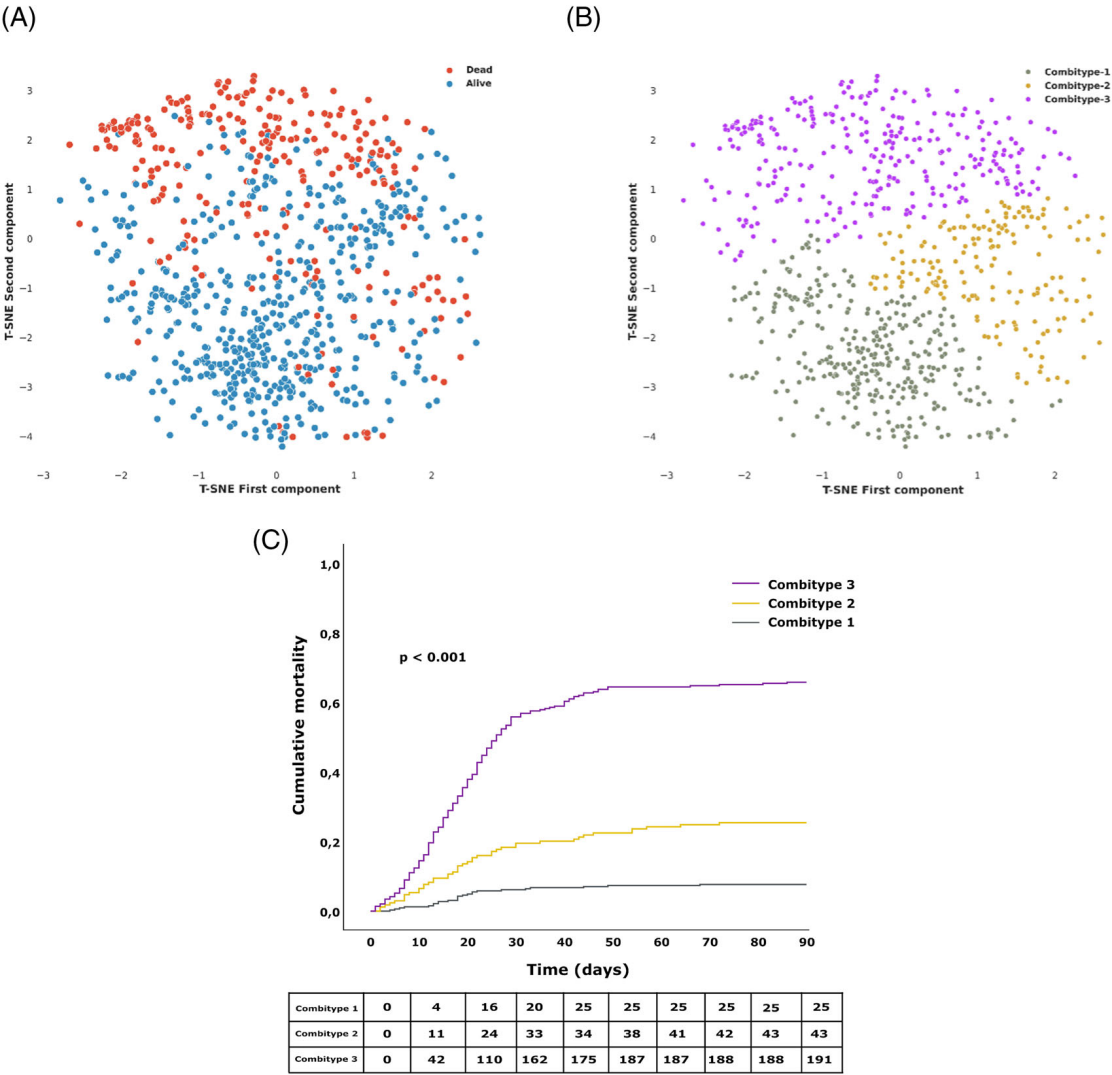
90‐day mortality groups (A) and partitional clustering groups (B) visualized using t‐Distributed Stochastic Neighbor Embedding. (C) Kaplan–Meier curves show the cumulative probability of mortality in the first 90 days following ICU admission. T‐SNE, t‐Distributed Stochastic Neighbor Embedding, *p*‐value, level of significance.

The three groups of 90‐day mortality risk exhibited different biological characteristics (Figure 3 and Table S2). The Combitype‐1 group had the lowest viral RNA load in plasma, the lowest prevalence of antigenemia, the highest concentration of anti‐SARS‐CoV‐2 S IgG and IgM antibodies, and a homeostatic response to infection, with reduced levels of all pro‐inflammatory cytokines and chemoattractant proteins tested (except RANTES). Thus, Combitype‐1 could be considered a group of “elite” viral controllers within the population of patients admitted to the ICU.

**FIGURE 3:**
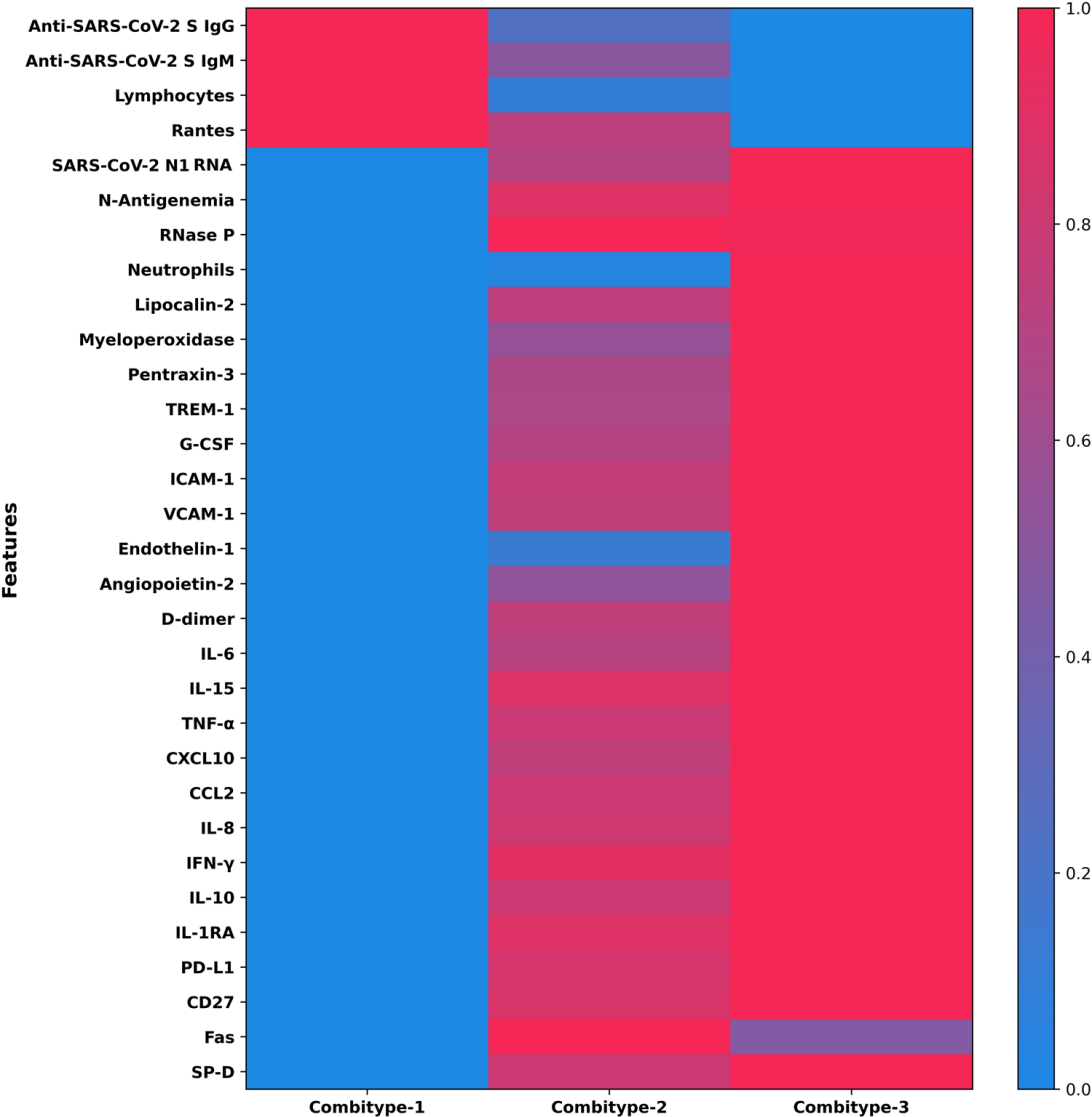
Heat map showing the three distinct patterns of inflammatory immune response by 90‐day mortality risk group (combitypes). Abbreviations: SARS‐CoV‐2, severe acute respiratory syndrome coronavirus 2; IgM, immunoglobulin M; IgG, immunoglobulin G; RANTES, regulated on activation normal T‐cell expressed and secreted protein; TREM‐1, triggering receptor expressed on myeloid cells; G‐CSF, granulocyte colony‐stimulating factor; ICAM‐1, intercellular adhesion molecule 1; VCAM‐1, vascular cell adhesion molecule 1; IL, interleukin; TNF, tumour necrosis factor; CXCL‐10, C‐X‐C motif chemokine ligand 10; CCL2, chemokine (C‐C motif) ligand 2; IFN, interferon; PD‐L1, programmed death‐ligand 1; CD‐27, cluster differentiation 27 molecule; SP‐D, surfactant protein D.

In contrast, the Combitype‐2 and ‐3 groups had a higher viral RNA load and higher prevalence of SASR‐CoV‐2 N antigen in plasma. The overall biomarker profile in the Combitype‐2 and Combitype‐3 groups indicated a broad dysregulation of the host response to infection, but with striking differences between these two groups. While the Combitype‐2 had moderate viral RNA load along with intermediate levels of inflammatory and endothelial dysfunction biomarkers, the Combitype‐3 showed the highest concentration in plasma of lipocalin‐2, MPO, VCAM‐1, PTX‐3, IL‐10, CXCL10, angiopoietin‐2, IL‐6, IL‐15, endothelin‐1, IL‐8, and TREM‐1, indicating an exacerbated pro‐inflammatory profile coupled with higher endothelial dysregulation and very high viral RNA load in plasma.

These three immune signatures were linked to significant clinical differences (Table 1). Patients in the Combitype‐1 group were younger and presented better respiratory function (PaO_2_/FiO_2_ ratio), and lower organ dysfunction (SOFA score) at ICU admission, together with lower frequency of hypertension, diabetes, chronic kidney disease, and chronic neurological disease as comorbidities. On the contrary, the Combitype‐3 group had the highest prevalence of diabetes and immunosuppression. In terms of complications during hospital admission, the Combitype‐1 group needed less often invasive mechanical ventilation and showed a lower frequency of secondary infections, acute kidney injury and septic shock, while the Combitype‐3 group suffered more frequently acute liver failure, acute kidney injury, coagulation disorders and septic shock. As mentioned earlier, the Combitype‐3 group was the one who presented the highest levels of viral RNA load and pro‐inflammatory mediators. Taken together, these results point to the important role of uncontrolled viral replication in the development of multiorgan failure and the extremely high mortality rate observed in this group. In line with these results, a previous investigation has shown a novel mechanism for propagating inflammation, which involves SARS‐CoV‐2 fragments,^9^ which could underlie the extrapulmonary pathologies observed in critical COVID‐19 patients, particularly in the Combitype‐3 group, which exhibited a very high SARS‐CoV‐2 RNA load in plasma.

**TABLE 1:** Clinical characteristics of the three groups of 90‐day mortality risk established according to partitional clustering analysis.

	Combitype‐1	Combitype‐2	Combitype‐3	*p*‐value (1 vs. 2)	*p*‐value (1 vs. 3)	*p*‐value (2 vs. 3)
**No. (%)**	326 (41.5)	169 (21.5)	290 (36.9)			
**Age (years)**	61.0 [51.0–69.0]	66.0 [58.0–73.0]	68.5 [61.0–75.0]	**<0.001**	**<0.001**	**0.007**
**Sex (female)**	105 (32.2)	46 (27.2)	90 (31.0)	0.673	0.821	0.673
**Days since symptoms onset**	10.0 [8.0–13.0]	10.0 [7.0–12.0]	9.0 [7.0–13.0]	**0.037**	**0.037**	0.767
**Epidemic period**						
**16 Mar. to 21 Jun., 2020**	34 (10.4)	10 (5.9)	13 (4.5)	0.237	**0.020**	0.746
**22 Jun. to 6 Dec., 2020**	200 (61.3)	102 (60.4)	173 (59.7)			
**7 Dec. 2020 to 27 Feb. 2021**	92 (28.2)	57 (33.7)	104 (35.9)			
**SOFA**	4.0 [3.0 ‐ 6.0]	5.5 [4.0 ‐ 7.2]	5.0 [4.0 ‐ 8.0]	**<0.001**	**<0.001**	0.940
**PaO_2_/FiO_2_ ratio**	119.1 [85.0 ‐ 164.4]	97.7 [76.7 ‐ 136.0]	100.0 [72.6 ‐ 134.8]	**0.001**	**0.001**	0.876
**Comorbidities**						
Hypertension	145 (44.5)	95 (56.2)	177 (61.2)	**0.026**	**<0.001**	0.337
Obesity	132 (40.5)	61 (36.1)	84 (29.1)	0.393	**0.012**	0.218
Diabetes	53 (16.3)	45 (26.6)	112 (38.6)	**0.012**	**<0.001**	**0.012**
Chronic pulmonary disease	32 (9.8)	22 (13.0)	47 (16.2)	0.431	0.074	0.431
Chronic cardiac disease	35 (10.7)	18 (10.7)	47 (16.2)	>0.999	0.182	0.207
Chronic kidney disease	9 (2.8)	14 (8.3)	37 (12.8)	**0.006**	**<0.001**	0.138
Chronic neurological disease	13 (4.0)	18 (10.7)	27 (9.3)	**0.004**	**0.007**	0.641
Immunosuppression	22 (6.7)	10 (5.9)	36 (12.4)	0.721	**0.016**	**0.025**
**Treatments**						
Hydroxychloroquine	36 (11.0)	9 (5.3)	11 (3.8)	**0.036**	**0.004**	0.601
Lopinavir/ritonavir	28 (8.6)	10 (5.9)	21 (7.3)	0.71	0.71	0.71
Tocilizumab	87 (26.7)	35 (20.7)	72 (25.0)	0.528	0.701	0.528
Remdesivir	65 (19.9)	32 (18.9)	41 (14.2)	0.883	0.236	0.350
**Complications**						
Invasive mechanical ventilation	208 (64.0)	155 (91.7)	262 (90.3)	**<0.001**	**<0.001**	0.746
Hyperglycaemia	226 (69.3)	132 (78.6)	245 (84.5)	0.057	**<0.001**	0.141
Secondary infections	136 (41.7)	110 (66.7)	176 (62.4)	**<0.001**	**<0.001**	0.422
Acute kidney injury	56 (17.2)	53 (31.4)	150 (51.7)	**<0.001**	**<0.001**	**<0.001**
Acute liver failure	74 (22.7)	41 (24.3)	101 (34.8)	0.781	**0.004**	**0.036**
Coagulation disorders	86 (26.4)	42 (24.9)	124 (42.8)	0.713	**<0.001**	**<0.001**
Septic shock	23 (9.8)	26 (22.4)	110 (50.7)	**0.001**	**<0.001**	**<0.001**
**Outcomes**						
UCI stay among survivors (days)	9 (5.0–19.0)	22 (11.0–36.0)	22 (10.0–44.0)	<0.001	<0.001	1.000
Hospital stay since ICU admission among survivors (days)	18 (12.0–35.0)	33 (22.0–58.0)	35 (24.0–59.0)	<0.001	<0.001	1.000
**90‐day Mortality**	25 (7.7)	43 (25.4)	191 (65.9)	**<0.001**	**<0.001**	**<0.001**

Statistics: Values are expressed as median (quartile 1 ‐ quartile 3) for continuous variables and absolute count (percentage) for categorical variables. Kruskal–Wallis test with False Discovery Rate adjustment was used to compare continuous variables, and Pearson's Chi‐square (𝛘^2^) or Fisher's exact tests were used to compare categorical variables. Significant differences (*p*‐value < 0.05) are shown in bold. Immunosuppression was a composed variable obtained from the summation of hematologic comorbidity, active malignant neoplasia, HIV (human immunodeficiency virus) infection or AIDS (acquired immune deficiency), solid organ transplantation and bone marrow transplantation.

Abbreviations: PaO_2_/FiO_2_, ratio of arterial oxygen partial pressure (PaO_2_ in mmHg) to fractional inspired oxygen (FiO2). Missing data were present for Days since symptoms onset (7), SOFA (15), PaO_2_/FiO_2_ ratio (103), Hypertension (1), Obesity (1), Chronic cardiac disease (1), Chronic kidney disease (1), Immunosuppression (1), Hydroxychloroquine (2), Lopinavir/ritonavir (2), Tocilizumab (2), Remdesivir (2), Invasive mechanical ventilation (1), Hyperglycaemia (1) and Septic shock (212).; *p*‐value, level of significance; SOFA, Sequential Organ Failure Assessment.

In conclusion, this is the first study combining SARS‐CoV‐2 RNA levels with host response data to develop a 90‐day mortality prediction model by an XGBoost algorithm and employing SHAP values to evaluate the influence of each biological feature on the outcome variable. Our results showed that SARS‐CoV‐2 RNA load was the most important biological factor influencing 90‐day mortality among COVID‐19 patients admitted to the ICU and revealed that endothelin‐1 and IL‐15 had a higher influence on COVID‐19 mortality than other pro‐inflammatory cytokines, like IL‐6. This prediction model confirmed our previous findings demonstrating that viral N1 RNA load was a predictor of 90‐day mortality.^4^ However, the current clustering analysis considering 33 biological features on top to viral RNA load enabled better classification of patients with different severity (Figure 4), revealing the existence of the group showing a better prognosis within critically ill COVID‐19 patients, the “elite” viral controllers. This group represented the largest group of our cohort and exhibited a robust antibody response that prevent uncontrolled viral replication and/or propagation, leading to more homeostatic immune responses to infection and increased survival. These results could help to understand the factors leading to survival not only in severe SARS‐CoV‐2 infection, but also in the infections caused by other emerging viruses.

**FIGURE 4:**
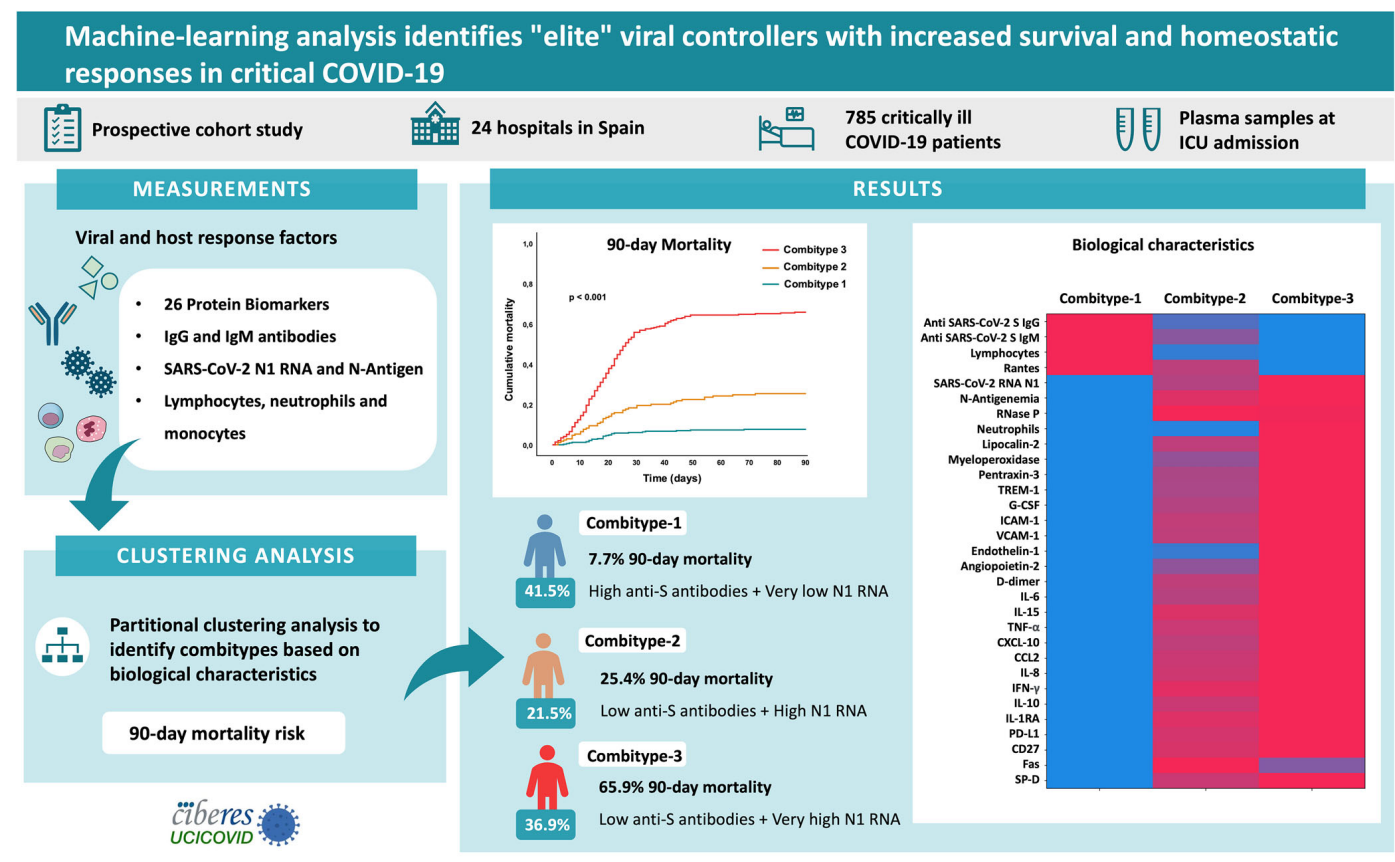
Machine learning analysis combining host‐response and virological data improves the characterization of subgroups of critically ill COVID‐19 patients with different prognosis. Abbreviations: SARS‐CoV‐2, severe acute respiratory syndrome coronavirus 2; IgM, immunoglobulin M; IgG, immunoglobulin G; RANTES, regulated on activation normal T‐cell expressed and secreted protein; TREM‐1, triggering receptor expressed on myeloid cells; G‐CSF, granulocyte colony‐stimulating factor; ICAM‐1, intercellular adhesion molecule 1; VCAM‐1, vascular cell adhesion molecule 1; IL, interleukin; TNF, tumour necrosis factor; CXCL‐10, C‐X‐C motif chemokine ligand 10; CCL2, chemokine (C‐C motif) ligand 2; IFN, interferon; PD‐L1, programmed death‐ligand 1; CD‐27, cluster differentiation 27 molecule; SP‐D, surfactant protein D.

## AUTHOR CONTRIBUTIONS

JFBM, APT, SR and AT participated in protocol development, study design and management. JFBM and SR participated in the analysis and interpretation of data. AAM developed the machine learning and statistical analysis and drafted the figures. NGM participated in the coordination of the clinical study, analyzed the data and wrote the manuscript. AO and TP developed the dPCR works and profiled the biomarkers. DVS participated in statistical analysis. LT, PRM, EBM, EGC, AUI, MCT, AE, SCF, IMV, FPG, LS, JLM, PVC, VSM, MGR, NC, MCMD, LJV, CML, RNJG, EM, ALV, WT, JAB, RHM, JB, PE, AMdG, CR, GA, GR, JBM, RC, MSV, EBP, EG, FC, MRN, JMSC, YPM, MTGU, MTBV, AMR, LPB, LRC, NAM, JMG, MLB, JC, CB, JG, MTN, JNdO, EPS, LGG, JCR and JME recruited the patients and collected the clinical data. SR, IM, MMV, MJMG, VM, MV and OC performed the antibody assays. LSR performed the extraction of viral RNA. APT and AdF analyzed the viral load data. NJ participated in profiling the biomarkers. DJK, FB and DdGC participated in the study design. AM, AC, LFB and RA participated in the study design and coordination. All authors have critically revised the manuscript and approved the final version. All authors agree to be accountable in ensuring that questions related to the accuracy or integrity of any part of the work are appropriately investigated and resolved. All authors confirm that they had full access to all the data in the study, verify the underlying data reported and accept responsibility to submit for publication.

## CONFLICT OF INTEREST STATEMENT

JFBM, AT, FB, RA, JME and APT have a patent application on SARS‐CoV‐2 antigenemia as a predictor of mortality in COVID‐19.

The remaining authors declare no conflicts of interest.

## ETHICAL APPROVAL

This is a sub‐study of the CIBERESUCICOVID study (NCT04457505), which received approval from the Institution's Internal Review Board (Comité Ètic d'Investigació Clínica, registry number HCB/2020/0370). Participant hospitals obtained the approval of the respective local ethics committee. The study was performed in full compliance with the Declaration of Helsinki and national and international law on data protection. Informed consent was obtained from each patient or legal representative.

## Supporting information



Supporting Information

## Data Availability

The data that support the findings of this study are available from the corresponding author upon reasonable request.
