# Supplementary material for: Machine‐learning analysis identifies “elite” viral controllers with increased survival and homeostatic responses in critical COVID‐19
Source: Clin Transl Med. 2025 Apr 25;15(5):e70241. doi: 10.1002/ctm2.70241 (PMC12031886; doi:10.1002/ctm2.70241)
Supplement: Supplementary file 1 — Supporting Information [file CTM2-15-e70241-s001.docx]

**SUPPLEMENTARY MATERIAL**

**Machine-learning analysis identifies "elite" viral controllers with increased survival and homeostatic responses in critical COVID-19**

**Methods**

**Study design and patients:** This is a re-analysis of the previously published cohort data from Bermejo-Martin *et al* [1]. 785 unvaccinated critically ill COVID-19 patients were included. The inclusion criteria were: (1) aged ≥ 18 years, (2) laboratory-confirmed SARS-CoV-2 infection and (3) a plasma-EDTA sample collected in the first 48 hours following admission to the ICU. All patients received corticosteroids treatment. Patients were recruited across 24 Spanish hospitals between March 17^th^ 2020 and February 27^th^ 2021, during the three epidemic periods of the pandemic as defined by the Spanish National Centre of Epidemiology [2]. Epidemic period 1 extended from March 16 to June 21, 2020, with B.1 being the most prevalent lineage during this period. Epidemic period 2 spanned from June 22 to December 6, 2020, with B.1.177 as the predominant lineage. Epidemic period 3 was from December 7, 2020, to February 27, 2021, with B.1.1.7 (Alpha) as the dominant variant [3]

Data on demographics, comorbidities, and previous treatment were recorded. The variable “Sex” corresponded to the sex assigned at birth. Standard laboratory and clinical data were collected at ICU admission. The pharmacologic treatments administered and interventions performed during hospitalization were also collected. Main complications during hospital stay were reported, including pulmonary complications, hyperglycaemia (glucose levels > 126 mg/dL), secondary infections, gastrointestinal bleeding, acute kidney injury (defined as any of: an increase in serum creatinine by ≥ 0·3 mg/dL within 48 hours, an increase in serum creatinine to ≥1·5 times baseline, and/or a urine volume <0·5 mL/kg/hour for 6 hours), acute hepatic failure (defined as any of: clinical jaundice, hyperbilirubinemia (i.e., blood total bilirubin level twice the upper limit of the normal range (>2·4mg/dL), and/or an increase in alanine transaminase or aspartate transaminase that is twice the upper limit of the normal range ( >80 U/L)) and anaemia defined by haemoglobin levels < 13 g/dL in male patients or < 12 g/dL in female patients. All data were pseudonymized and stored in a REDCap database hosted in the Centro de Investigación Biomédica en Red (CIBER), Madrid, Spain. Further details on the study methodology were published elsewhere [4].

**Blood samples:** Blood samples were collected in ethylenediaminetetraacetic acid (EDTA) tubes and immediately centrifuged to separate plasma. Plasma was stored at -80ºC at the participant sites, including the IRB-Lleida Biobank 119 (B.0000682) / “Plataforma Biobancos PT17/0015/0027", The Hospital Clinic Biobank in Barcelona and the National DNA Bank and the Hospital Universitario de Salamanca Biobank (both in Salamanca). Frozen plasma samples were sent to the BioSepsis laboratory in Valladolid, Spain, for viral load quantification and biomarker profiling. An aliquot was sent to the National Centre of Microbiology for antibody quantification.

**Quantification of SARS-CoV-2 N1 and human ribonuclease P (RNAse P) RNAs in plasma:** SARS-CoV-2 RNA was extracted from 140 µl of plasma using the QIAamp® Viral RNA Mini Kit (Qiagen, Venlo, Netherlands), according to manufacturer instructions. Quantification of the SARS-COV-2 Nucleocapsid Region 1 (N1) and RNAse P RNA was performed in five μl of the eluted solution using the Bio-Rad SARS-CoV-2 ddPCR kit according to manufacturer’s specifications on a QX-200 droplet digital PCR platform from the same provider.

**N-antigenemia profiling: N-**antigenemia was defined as a positive result for the presence of N-antigen of SARS-CoV-2 in plasma by using the Panbio® COVID-19 Ag Rapid Test (Abbott, Chicago, IL, USA) as previously described [5].

**Antibody concentration and function evaluation:** A specific immunoassay was developed to quantify anti-SARS-CoV-2 S IgG and IgM antibodies in plasma, as previously described [1].

**Biomarker profiling:** 26 biomarkers were quantified in plasma using the Ella-SimplePlex^TM^ system (Bio-Techne, Minneapolis, MN, USA) as per manufacturer instructions: lipocalin-2, myeloperoxidase, pentraxin 3 (PTX-3), triggering receptor expressed on myeloid cells (TREM-1), Granulocyte colony-stimulating factor (G-CSF) (granulocyte biology); intercellular adhesion molecule 1 (ICAM-1), Vascular Cell Adhesion Molecule 1 (VCAM-1), endothelin-1, angiopoietin 2 (endothelial dysfunction); D-dimer (coagulation); interleukin (IL)-6, IL-15, Tumour Necrosis factor alpha (TNF-α) (inflammation); C-X-C motif chemokine ligand 10 (CXCL10), C-C Motif Chemokine Ligand 2 (CCL2), IL-8, regulated on activation, normal T cell expressed and secreted protein (RANTES) (chemotaxis); IFN-γ (interferon response); Programmed Death-ligand 1 (B7-H1/PD-L1), IL-10, IL-1ra (immunosuppression); IL-7, cluster differentiation 27 molecule (CD27) (T-cell biology); FAS (apoptosis); SPD (surfactant protein D) and EGF (epithelial growth factor).

**Outcome and predictor variables:** the outcome was all-cause mortality in the first 90 days following admission to the ICU. The predictor variables included in the analysis were 26 laboratory plasma biomarkers (pg/ml) evaluated by SimplePlex-ELLA (see above), SARS-CoV-2 RNA N1 (copies/mL), RNase P (copies/mL), anti-SARS-CoV-2 S IgG (AUC), anti-SARS-CoV-2 S IgM (AUC), lymphocytes (cells/ml), neutrophils (cells/ml), monocytes (cells/ml) and N-antigenemia (categorical variable, % of positive patients).

**eXtreme Gradient Boosting (XGBoost) modelling and partitional clustering:** in the database with 785 patients, two approaches were used to treat missing values: i) missing values were imputed using the mode for categorical variables and the median for continuous variables, and also were treated with the multivariate imputation of chained equations (MICE) algorithm; ii) no specific treatment on missing data was done, but the XGBoost algorithm handled the missing as a value. Subsequently, both approaches models were tested with and without imputation, and finally, the data set of 785 patients without imputation was chosen because it was the best performing. This data set was randomly divided into one for training (80%) and another for independent testing (20%). Plasma biomarker values were ln-transformed.

An integrated explanatory framework model was developed using the Python package XGBoost in Python 3.11.3 (Python Software Foundation, Oregon, USA) to quantify the contribution of features influencing the outcome variable in the predictive model. As a previous analysis step, we performed a one-hot encoded in categorical features, a standard process in machine learning (ML) techniques. The model hyperparameters were tuned using GridSearchCV ([^1^](https://scikit-learn.org/stable/modules/generated/sklearn.model_selection.GridSearchCV.html)) to optimize the area under the receiver operating characteristic curve (AUROC), with a five-fold cross-validation to validate model performance and minimize the likelihood of overfitting. The final model was constructed with specific hyperparameters, including 300 trees, a learning rate of 0.01, a maximum depth of 4 and a subsample of 0.6 as hyperparameters. To obtain explanations of the features that drive patient-specific predictions, we used a Shapley additive explanation (SHAP) algorithm. The SHAP algorithm provides an explanation of 90-day mortality. In addition, we characterized the 90-day mortality using a partitional clustering algorithm (K-means). This multidimensional learning methodology groups n observations or characteristics into K groups (clusters), where intragroup observations are similar and intergroup observations are more distinct. The optimal number of clusters was determined using the NbClust package for R [6]. This package assessed 26 different internal validation metrics, considering both cluster compactness and separation. Based on voting system, the package determined that K=3 was the most suitable number of clusters.

To facilitate visualization of the K groups, we applied the t-Distributed Stochastic Neighbor Embedding (t-SNE), a powerful technique for reducing high-dimensional data to lower dimensions. Patients were then categorized into three distinct combitypes based on their biological characteristics (Combitype-1, Combitype-2 and Combitype-3). To assess the variability in 90-day survival explained by the three combitypes, we used Nagelkerke´s Pseudo R^2^, a metric that measures the proportion of variation in the outcome explained by the model with the combitypes compared to a null model. In this case, we obtained a Pseudo R^2^ of 95%, indicating that the combitypes explain a significant portion of the observed variability and survival.

**Statistical analysis:** Differences between groups were assessed using Pearson's Chi-square test (χ2) or Fisher's exact test for categorical variables and the Kruskal–Wallis test or Mann-Whitney U test for continuous ones. Multiple testing corrections were made using the Benjamini and Hochberg procedure (*q*-value). Statistical analysis was performed using IBM SPSS Statistics 25.0 (SPSS INC, Armonk, NY, U.S.A). The level of significance was set at 0·05.

**References:**

1. Bermejo-Martin JF, García-Mateo N, Motos A, et al (2023) Effect of viral storm in patients admitted to intensive care units with severe COVID-19 in Spain: a multicentre, prospective, cohort study. Lancet Microbe 4:e431–e441. https://doi.org/10.1016/S2666-5247(23)00041-1

2. Informe n^o^ 87. Situación de COVID-19 en España a 14 de julio de 2021. https://repisalud.isciii.es/bitstreams/788517eb-101c-4890-87a4-74ab2d38cbdb/download. Accessed 20 Jan 2025

3. Troyano-Hernáez P, Reinosa R, Holguín Á (2022) Evolution of SARS-CoV-2 in Spain during the First Two Years of the Pandemic: Circulating Variants, Amino Acid Conservation, and Genetic Variability in Structural, Non-Structural, and Accessory Proteins. Int J Mol Sci 23:6394. https://doi.org/10.3390/ijms23126394

4. Torres A, Motos A, Ceccato A, et al (2022) Methodology of a Large Multicenter Observational Study of Patients with COVID-19 in Spanish Intensive Care Units. Arch Bronconeumol 58 Suppl 1:22–31. https://doi.org/10.1016/j.arbres.2022.03.010

5. Almansa R, Eiros JM, de Gonzalo-Calvo D, et al (2022) N-antigenemia detection by a rapid lateral flow test predicts 90-day mortality in COVID-19: a prospective cohort study. Clin Microbiol Infect Off Publ Eur Soc Clin Microbiol Infect Dis S1198-743X(22)00282–8. https://doi.org/10.1016/j.cmi.2022.05.023

6. Charrad M, Ghazzali N, Boiteau V, Niknafs A (2014) NbClust: An R Package for Determining the Relevant Number of Clusters in a Data Set. J Stat Softw 61:1–36. https://doi.org/10.18637/jss.v061.i06

**Supplementary Table 1**. **Biological characteristics of the patients admitted to the intensive care unit.**

|  | Missing data | Overall | 90-day survivors | 90-day non-survivors | p-value |
| --- | --- | --- | --- | --- | --- |
| No. (%) |  | **785** | **526 (67.00)** | **259 (33.00)** |  |
| Measurements at ICU admission |  |  |  |  |  |
| N-Antigenemia (n, %) | 35 | 325 (43.3) | 204 (40.5) | 121 (49.2) | **0.024** |
| SARS-CoV-2 RNA N1 (copies/mL) | 25 | 980.8 (153.7 – 5019.1) | 613.7 (104.0 – 2746.4) | 2857.0 (403.1 – 11561.4) | **< 0.001** |
| RNase P (copies x 10^3^/mL) | 25 | 76.4 (38.2 – 143.3) | 69.0 (32.6 – 128.5) | 97.4 (52.2 – 197.7) | **< 0.001** |
| Lymphocytes (cells x 10^3^/ml) | 39 | 0.7 (0.4 – 0.9) | 0.7 (0.5 – 1.0) | 0.6 (0.4 – 0.8) | **0.007** |
| Neutrophils (cells x 10^3^/ml) | 58 | 8.3 (5.8 – 11.7) | 7.9 (5.7 – 10.8) | 9.7 (6.2 – 12.8) | **< 0.001** |
| Monocytes (cells x 10^3^/ml) | 64 | 0.4 (0.2 – 0.6) | 0.4 (0.2 – 0.6) | 0.4 (0.2 – 0.6) | 0.260 |
| Anti-SARS-CoV-2 S IgG (AUC) | 1 | 191.5 (21.6 – 952.0) | 210.2 (31.2 - 837.2) | 156.1 (6.9 – 1226.0) | 0.485 |
| Anti-SARS-CoV-2 S IgM (AUC) | 1 | 43.5 (2.0 – 164.1) | 43.1 (5.1 - 166.4) | 46.0 (0.0 – 160.1) | 0.141 |
| Biomarkers at ICU admission |  |  |  |  |  |
| ICAM-1 (ng/mL) | 22 | 426.2 (349.4 – 529.2) | 410.4 (343.5 – 498.4) | 463.2 (374.5 – 566.6) | **< 0.001** |
| Lipocalin-2 (ng/mL) | 22 | 98.4 (74.4 – 135.1) | 93.4 (72.7 – 126.3) | 112.8 (83.2 – 178.5) | **< 0.001** |
| Myeloperoxidase (ng/mL) | 22 | 178.5 (105.4 – 308.9) | 161.5 (95.7 – 264.3) | 223.6 (128.5 – 443.9) | **< 0.001** |
| VCAM-1 (ng/mL) | 22 | 1270.9 (961.6 – 1645.2) | 1198.2 (903.7 – 1551.3) | 1449.6 (1067.2 – 1893.1) | **< 0.001** |
| D-dimer (ng/mL) | 29 | 1908.9 (1056.4 – 4104.8) | 1674.3 (983.0- 3443.2) | 2580.2 (1302.6 – 6291.1) | **< 0.001** |
| Rantes (ng/mL) | 29 | 23.7 (13.1 – 42.5) | 25.9 (15.4 – 45.9) | 18.6 (9.0 – 34.4) | **< 0.001** |
| Fas (ng/mL) | 28 | 14.6 (11.2 – 19.2) | 14.4 (10.9 – 18.4) | 15.1 (11.5 – 20.7) | **0.013** |
| CD27 (ng/mL) | 28 | 8.0 (5.7 – 11.8) | 7.5 (5.4 – 10.8) | 9.3 (6.3 – 14.8) | **< 0.001** |
| SP-D (ng/mL) | 17 | 18.3 (10.1 – 35.7) | 16.8 (9.6 – 31.1) | 22.6 (11.0 – 57.2) | **< 0.001** |
| PTX-3 (ng/mL) | 29 | 29.9 (13.1 – 65.5) | 26.2 (11.5 – 56.8) | 42.1 (19.4 – 89.0) | **< 0.001** |
| IL-10 (pg/mL) | 17 | 12.9 (7.4 – 23.0) | 11.0 (6.5 – 19.6) | 17.2 (10.5 – 28.0) | **< 0.001** |
| IL-7 (pg/mL) | 17 | 6.8 (4.3 – 11.0) | 6.9 (4.4 – 11.1) | 6.5 (3.8 – 11.0) | 0.160 |
| CXCL10 (ng/mL) | 18 | 1.6 (0.9 – 2.5) | 1.5 (0.8 – 2.3) | 1.9 (1.2 – 2.8) | **< 0.001** |
| Angiopoietin-2 (ng/mL) | 17 | 1.2 (0.8 – 1.9) | 1.1 (0.7 – 1.7) | 1.3 (0.8 – 2.2) | **< 0.001** |
| IL-1RA (ng/mL) | 24 | 0.9 (0.6 – 1.7) | 0.9 (0.6 – 1.6) | 1.0 (0.6 – 1.9) | **0.003** |
| IL-6 (pg/mL) | 17 | 47.4 (17.1 – 175.5) | 38.5 (13.4 – 150.0) | 60.7 (27.6 – 337.5) | **< 0.001** |
| CCL2 (pg/mL) | 17 | 406.5 (241.0 – 743.3) | 356.0 (218.0 – 613.0) | 566.0 (308.0 – 961.0) | **< 0.001** |
| IL-15 (pg/mL) | 17 | 6.3 (4.8 – 8.2) | 5.8 (4.7 – 7.4) | 7.6 (5.5 – 10.1) | **< 0.001** |
| IFN-γ (pg/mL) | 17 | 1.9 (0.8 – 5.2) | 1.8 (0.7 – 4.8) | 1.9 (0.9 – 6.0) | 0.102 |
| TNF-α (pg/mL) | 17 | 11.2 (8.6 – 15.3) | 10.8 (8.4 – 14.5) | 12.6 (9.2 – 17.2) | **< 0.001** |
| Endothelin-1 (pg/mL) | 28 | 2.3 (1.5 – 3.4) | 2.0 (1.4 – 2.9) | 3.0 (2.1 – 4.4) | **< 0.001** |
| IL-8 (pg/mL) | 28 | 18.2 (11.7 – 26.5) | 16.1 (10.8 – 22.9) | 22.0 (16.0 – 33.5) | **< 0.001** |
| EGF (pg/mL) | 31 | 34.2 (14.1 – 81.4) | 37.2 (16.6 – 86.8) | 27.6 (10.3 – 76.4) | **0.009** |
| TREM-1 (pg/mL) | 32 | 604.0 (423.0 – 816.5) | 559.0 (389.5 – 738.5) | 716.0 (527.0 – 1063.8) | **< 0.001** |
| PD-L1 (pg/mL) | 10 | 211.0 (161.0 – 290.0) | 201.0 (154.5 – 268.5) | 237.0 (176.5 – 327.0) | **< 0.001** |
| G-CSF (pg/mL) | 11 | 47.8 (28.7 – 85.7) | 47.2 (28.8 – 81.2) | 49.4 (28.3 – 90.5) | 0.398 |

Statistics: Continuous variables are represented as median (quartile 1 - quartile 3) and categorical variables as absolute count (n, (%). P-values were assessed by using the U Mann-Whitney statistical test for continuous variables and the Chi-squared test for categorical variables. Significant differences (p-value < 0.05) are shown in bold. Abbreviations: p-value, level of significance; SOFA, Sequential Organ Failure Assessment; SARS-CoV-2, severe acute respiratory syndrome coronavirus 2; IgM, Immunoglobulin M; IgG, Immunoglobulin G; ICAM-1, intercellular adhesion molecule 1; VCAM-1, vascular cell adhesion molecule 1; RANTES, regulated on activation, normal T-cell expressed and secreted protein; CD-27, cluster differentiation 27 molecule; SP-D, surfactant protein D; PTX-3, pentraxin 3; IL, interleukin; CXCL-10, C-X-C motif chemokine ligand 10; CCL2, chemokine (C-C motif) ligand 2; IFN, interferon; TNF, tumor necrosis factor; EGF, epithelial growth factor; TREM-1, triggering receptor expressed on myeloid cells 1; PD-L1, programmed death-ligand 1; G-CSF, granulocyte colony-stimulating factor.

**Supplementary Figure 1. ROC curve to classify patients with 90-day mortalit**y.


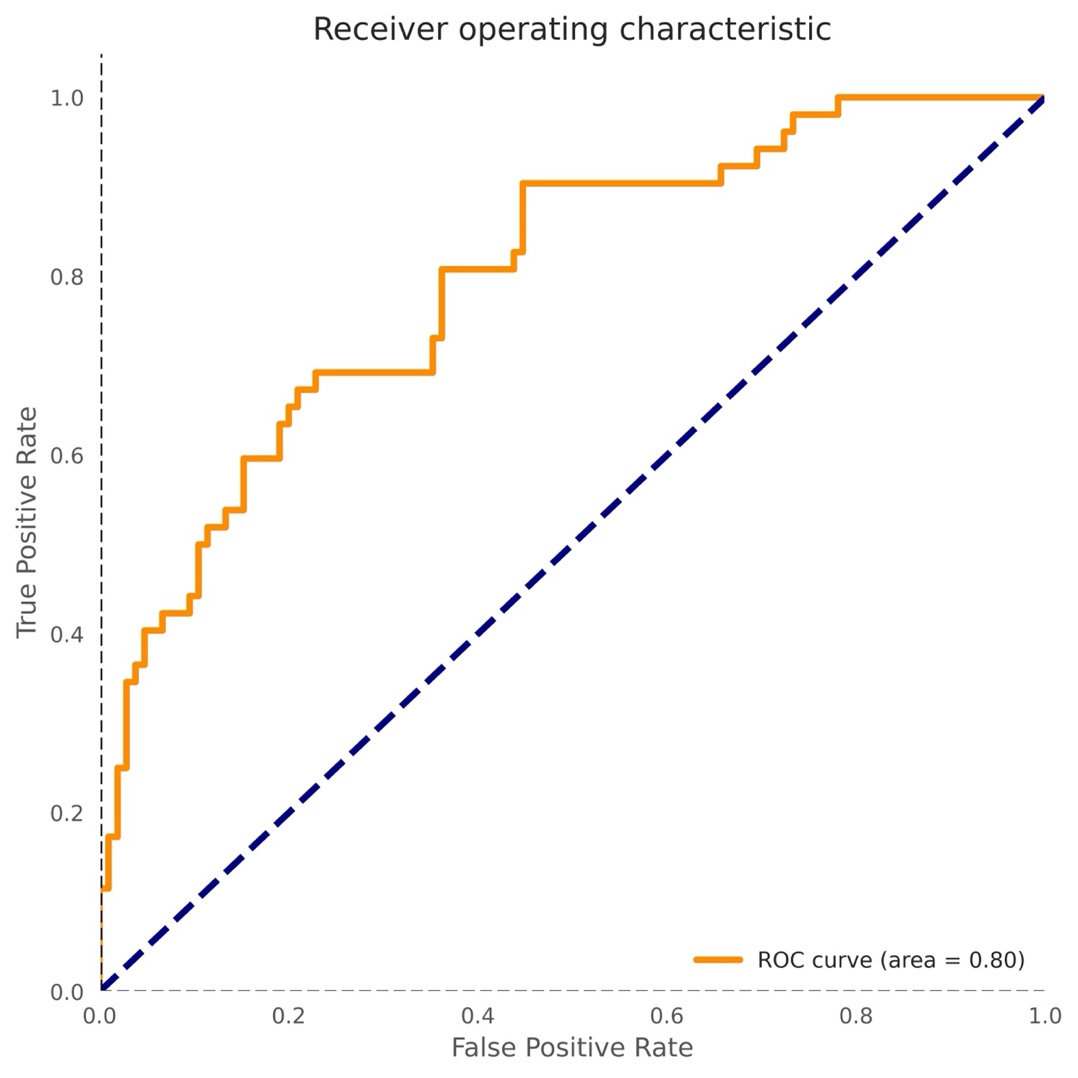


Abbreviations: ROC, receiver operating characteristic; AUROC, area under the receiver operating characteristic curve.

**Supplementary Table 2. Biological characteristics of the three groups of 90-day mortality risk established according to partitional clustering analysis.**

|  | **Combitype-1** | **Combitype-2** | **Combitype-3** | **p-value (1 *vs.* 2)** | **p-value (1 *vs.* 3)** | **p-value (2 *vs.* 3)** |
| --- | --- | --- | --- | --- | --- | --- |
| **No. (%)** | 326 (41.5) | 169 (21.5) | 290 (36.9) |  |  |  |
| **Measurements at ICU admission** |  |  |  |  |  |  |
| N-Antigenemia | 81 (26.1) | 83 (52.2) | 161 (57.3) | **<0.001** | **<0.001** | 0.351 |
| SARS-CoV-2 RNA N1 (copies/mL) | 165.9 (0.0 - 739.8) | 1730.4 (311.7 - 6509.3) | 4813.9 (1198.6 - 13741.7) | **<0.001** | **<0.001** | **<0.001** |
| RNase P (copies x 10^3^/mL) | 51349.2 (26091.2 - 95323.8) | 104971.6 (47888.6 - 157996.8) | 103434.7 (53444.8 - 210482.0) | **<0.001** | **<0.001** | 0.322 |
| Anti-SARS-CoV-2 S IgG (AUC) | 381.0 (72.9 - 1159.0) | 118.2 (8.5 - 873.7) | 81.0 (2.8 - 612.0) | **<0.001** | **<0.001** | 0.237 |
| Anti-SARS-CoV-2 S IgM (AUC) | 58.7 (10.0 - 188.4) | 31.3 (0.0 - 196.2) | 16.8 (0.0 - 103.8) | **0.009** | **<0.001** | 0.145 |
| Lymphocytes (cells x 10^3^/ml) | 0.7 (0.5 - 1.1) | 0.6 (0.4 - 0.9) | 0.6 (0.4 - 0.9) | **0.011** | **<0.001** | 0.531 |
| Neutrophils (cells x 10^3^/ml) | 7.7 (5.7 - 10.9) | 7.8 (5.9 - 10.6) | 9.7 (6.0 - 12.8) | 0.891 | **0.001** | **0.01** |
| Monocytes (cells x 10^3^/ml) | 0.4 (0.2 - 0.6) | 0.3 (0.2 - 0.5) | 0.4 (0.2 - 0.6) | 0.158 | 0.158 | 0.982 |
| **Biomarkers** |  |  |  |  |  |  |
| ICAM-1 (ng/mL) | 389.4 (323.2 – 476.8) | 441.5 (365.2 – 539.5) | 459.3 (383.0 – 563.7) | **<0.001** | **<0.001** | 0.192 |
| Lipocalin-2 (ng/mL) | 83.5 (66.5 – 107.4) | 108.3 (85.9 – 135.6) | 118.4 (85.2 – 179.4) | **<0.001** | **<0.001** | **0.029** |
| Myeloperoxidase (ng/mL) | 128.4 (82.0 – 212.3) | 183.8 (129.0 – 268.4) | 244.6 (132.2 – 477.6) | **<0.001** | **<0.001** | **<0.001** |
| VCAM-1 (ng/mL) | 1069.3 (822.7 – 1389.6) | 1368.3 (1041.3 – 1655.2) | 1486.9 (1109.1 – 1933.9) | **<0.001** | **<0.001** | **0.009** |
| D-dimer (ng/mL) | 1398.5 (858.2 – 2768.2.0) | 2137.7 (1186.6 – 4920.3) | 2462.6 (1421.2 – 5607.3) | **<0.001** | **<0.001** | 0.222 |
| Rantes (ng/mL) | 26.9 (15.5 – 46.4) | 24.8 (14.3 – 46.1) | 19.9 (10.6 – 38.0) | 0.303 | **<0.001** | **0.018** |
| Fas (ng/mL) | 13.6 (10420.0 - 17342.5) | 16.6 (12.7 – 21.1) | 14.9 (11.2 – 20.4) | **<0.001** | **0.001** | 0.057 |
| CD27 (ng/mL) | 6.8 (5.2 – 9.4) | 8.7 (6.1 – 12.7) | 9.1 (6.3 – 15.9) | **<0.001** | **<0.001** | 0.388 |
| SP-D (ng/mL) | 15.0 (9.3 – 25.5) | 21.3 (12.6 – 40.0) | 23.3 (11.0 – 51.8) | **<0.001** | **<0.001** | 0.564 |
| PTX-3 (ng/mL) | 19.1 (9.4 – 39.1) | 33.8 (13.6 – 72.0) | 45.2 (20.7 – 94.4) | **<0.001** | **<0.001** | **0.004** |
| IL-10 (pg/mL) | 7.8 (5.2 - 13.2) | 16.3 (9.7 - 24.9) | 19.5 (11.8 - 31.2) | **<0.001** | **<0.001** | **0.01** |
| IL-7 (pg/mL) | 6.8 (4.2 - 10.7) | 6.9 (4.8 - 10.7) | 6.6 (4.2 - 11.8) | 0.717 | 0.717 | 0.717 |
| CXCL10 (pg/mL) | 1032.5 (536.2 - 1565.2) | 1827.0 (1259.0 - 2644.0) | 2235.5 (1485.0 - 3168.7) | **<0.001** | **<0.001** | **0.002** |
| Angiopoietin-2 (pg/mL) | 1007.0 (690.7 - 1519.5) | 1196.0 (833.0 - 1750.0) | 1393.0 (852.0 - 2243.0) | **0.003** | **<0.001** | **0.029** |
| IL-1RA (pg/mL) | 702.5 (475.8 - 1138.5) | 1068.0 (650.5 - 2094.5) | 1124.0 (709.8 - 2148.0) | **<0.001** | **<0.001** | 0.352 |
| IL-6 (pg/mL) | 21.6 (9.5 - 85.6) | 52.6 (23.3 - 202.0) | 75.8 (29.3 - 453.0) | **<0.001** | **<0.001** | **0.009** |
| CCL2 (pg/mL) | 272.0 (171.3 - 442.8) | 499.0 (302.0 - 896.0) | 583.0 (316.0 - 1035.0) | **<0.001** | **<0.001** | 0.215 |
| IL-15 (pg/mL) | 5.1 (4.1 - 6.2) | 7.3 (5.6 - 8.6) | 7.6 (5.8 - 10.1) | **<0.001** | **<0.001** | **0.029** |
| IFN-γ (pg/mL) | 1.3 (0.6 - 3.0) | 2.4 (1.3 - 7.9) | 2.6 (1.0 - 8.0) | **<0.001** | **<0.001** | 0.774 |
| TNF-α (pg/mL) | 9.6 (7.0 - 12.6) | 12.3 (9.9 - 16.9) | 13.1 (9.7 - 17.9) | **<0.001** | **<0.001** | 0.424 |
| Endothelin-1 (pg/mL) | 1.7 (1.2 - 2.5) | 1.9 (1.5 - 2.3) | 3.4 (2.7 - 5.2) | **0.030** | **<0.001** | **<0.001** |
| IL-8 (pg/mL) | 12.8 (9.3 - 18.1) | 20.3 (13.9 - 29.8) | 22.5 (17.2 - 34.4) | **<0.001** | **<0.001** | **0.023** |
| EGF (pg/mL) | 37.2 (16.5 - 90.8) | 34.4 (12.7 - 77.3) | 29.4 (11.6 - 76.5) | 0.311 | 0.061 | 0.452 |
| TREM-1 (pg/mL) | 486.0 (350.0 - 609.0) | 649.5 (501.0 - 804.3) | 754.5 (565.5 - 1033.3) | **<0.001** | **<0.001** | **<0.001** |
| PD-L1 (pg/mL) | 174.5 (137.0 - 223.2) | 234.5 (191.8 - 300.2) | 247.0 (195.0 - 347.0) | **<0.001** | **<0.001** | 0.127 |
| G-CSF (pg/mL) | 40.9 (24.1 - 68.4) | 50.2 (32.2 - 96.6) | 54.9 (32.6 - 104.2) | **<0.001** | **<0.001** | 0.319 |

**Statistics**: Continuous variables are represented as median (quartile 1 - quartile 3) and categorical variables as absolute count (n, (%), differences were assessed by using the Kruskal–Wallis test with False Discovery Rate adjustment for continuous variables, and Chi-squared test for categorical variables. Significant differences (p-value < 0.05) are shown in bold. **Abbreviations:** p-value, level of significance; SARS-CoV-2, severe acute respiratory syndrome coronavirus 2; IgM, Immunoglobulin M; IgG, Immunoglobulin G; ICAM-1, intercellular adhesion molecule 1; VCAM-1, vascular cell adhesion molecule 1; RANTES, regulated on activation, normal T-cell expressed and secreted protein; CD-27, cluster differentiation 27 molecule; SP-D, surfactant protein D; PTX-3, pentraxin 3; IL, interleukin; CXCL-10, C-X-C motif chemokine ligand 10; CCL2, chemokine (C-C motif) ligand 2; IFN, interferon; TNF, tumor necrosis factor; EGF, epithelial growth factor; TREM-1, triggering receptor expressed on myeloid cells 1; PD-L1, programmed death-ligand 1; G-CSF, granulocyte colony stimulating factor.
